# State-of-the-Art Extraction Methodologies for Bioactive Compounds from Algal Biome to Meet Bio-Economy Challenges and Opportunities

**DOI:** 10.3390/molecules23112953

**Published:** 2018-11-12

**Authors:** Juan Eduardo Sosa-Hernández, Zamantha Escobedo-Avellaneda, Hafiz M. N. Iqbal, Jorge Welti-Chanes

**Affiliations:** Tecnologico de Monterrey, Escuela de Ingeniería y Ciencias, Centro de Biotecnología FEMSA, Ave. Eugenio Garza Sada 2501, C.P. 64849 Monterrey, N.L., Mexico; eduardo.sosa@itesm.mx (J.E.S.-H.); zamantha.avellaneda@itesm.mx (Z.E.-A.); jwelti@tec.mx (J.W.-C.)

**Keywords:** bioactive compounds, algal biome, extraction methodologies, enzyme-assisted extraction, supercritical-fluid extraction, microwave-assisted extraction, pressurized-liquid extraction, applications, anticancer, antibacterial, antiviral

## Abstract

Over the years, significant research efforts have been made to extract bioactive compounds by applying different methodologies for various applications. For instance, the use of bioactive compounds in several commercial sectors such as biomedical, pharmaceutical, cosmeceutical, nutraceutical and chemical industries, has promoted the need of the most suitable and standardized methods to extract these bioactive constituents in a sophisticated and cost-effective manner. In practice, several conventional extraction methods have numerous limitations, e.g., lower efficacy, high energy cost, low yield, etc., thus urges for new state-of-the-art extraction methodologies. Thus, the optimization along with the integration of efficient pretreatment strategies followed by traditional extraction and purification processes, have been the primary goal of current research and development studies. Among different sources, algal biome has been found as a promising and feasible source to extract a broader spectrum of bioactive compounds with point-of-care application potentialities. As evident from the literature, algal bio-products includes biofuels, lipids, polyunsaturated fatty acids, pigments, enzymes, polysaccharides, and proteins. The recovery of products from algal biomass is a matter of constant development and progress. This review covers recent advancements in the extraction methodologies such as enzyme-assisted extraction (EAE), supercritical-fluid extraction (SFE), microwave-assisted extraction (MAE) and pressurized-liquid extraction (PLF) along with their working mechanism for extracting bioactive compounds from algal-based sources to meet bio-economy challenges and opportunities. A particular focus has been given to design characteristics, performance evaluation, and point-of-care applications of different bioactive compounds of microalgae. The previous and recent studies on the anticancer, antibacterial, and antiviral potentialities of algal-based bioactive compounds have also been discussed with particular reference to the mechanism underlying the effects of these active constituents with the related pathways. Towards the end, the information is also given on the possible research gaps, future perspectives and concluding remarks.

## 1. Introduction

Biologically active constituents so-called “bioactive compounds” are typically available in various bio-based sources including algal biome and others though in different quantities with specific functionalities. Among bioactive compounds, carotenoids, tocopherols, tocotrienols, and others are of supreme interest. In past years, they have been extensively studied and used as a nutraceutical or functional constituents for several health purposes. In recent years, the research interest in bioactive compounds increased exponentially in the biomedical, pharmaceutical, cosmeceutical, nutraceutical and chemical industries. However, the use of bioactive compounds in different sectors implies the need and consequent demand most appropriate and standardized strategies of extraction [1]. The quantitative and qualitative characteristics of biologically active compounds strongly rely on the selection of an appropriate extraction method [1,2,3]. For instance, the state-of-the-art processes to extract polysaccharide and exopolysaccharide from algal-based biomass and the analytical strategies for their characterization have been reviewed [3]. A properly developed and exploited extraction methodology plays a significant and critical role in the quality of the end products. It is also equally important to consider various influencing factors such as physiochemical properties of the source material, matrix properties, solvent type and concentration, pH, temperature, pressure and time that can affect the overall performance of the extraction process [4]. The overall efficiency of the extraction methods mostly depends on the following points, i.e., (1) critical input parameters; (2) understanding the nature of the source; (3) interplay between the process and the source and (4) chemistry of bioactive compounds.

Owing to the extreme diversity and source-based variation in their physiochemical properties, a precise classification of bioactive compounds is not well established yet. Also, the classification is quite complicated because of certain similarities between molecules and/or dissimilarities between functionally and chemically interrelated compounds. However, they have been well categorized from different perspectives, i.e., based on: (1) taxonomic sources i.e., family and genus; (2) biosynthetic pathways; (3) their physical source i.e., plant or animal; (4) their unique structure including ring and linear structures; (5) available functional moieties; (6) precursor molecules; (7) sugar moiety; (8) chemical contents, etc. Croteau [5] divided bioactive compounds into three categories, i.e., (1) terpenes and terpenoids (approximately 25,000 types); (2) alkaloids (approximately 12,000 types); and (3) phenolic compounds (approximately 8000 types). From the biosynthetic pathways view, there are four major pathways, i.e., (1) shikimic acid pathway; (2) malonic acid pathway; (3) mevalonic acid pathway; and (4) non-mevalonate (MEP) pathway. The simplest routes for biosynthesis include glycosides and polysaccharides, synthesized from pentose; phenolic compounds, tannins, and alkaloids, synthesized from shikimic acid; phenolic compounds and alkaloids, synthesized from acetate-malonate; and terpenes, steroids, and alkaloids, synthesized from mevalonic acid pathway [6]. According to Dewick [6], a broader spectrum of secondary metabolites is biosynthetically formed after the intermediate acetyl coenzyme A (acetyl-CoA), shikimic acid, mevalonic acid, and 1-deoxyxylulose 5-phosphate.

Considering the value-added characteristics of bioactive compounds, herein, this review compiled salient information covering various industrially relevant aspects to meet current bio-economy challenges and opportunities. More specifically, this review covers recent advancements in the extraction methodologies such as EAE, SFE, MAE, and PLF, along with their working mechanism for extracting bioactive compounds from algal-based sources.

## 2. An Immense Source of Excellent Performance: Algal Biome

To equally fulfill the rising demand of naturally occurring bioactive compounds among all industrial sectors, researchers have regained their interests in natural sources such as algal biome “a treasure of untouched sources”. Algal biome belongs to the marine region of aquatic biome (the largest biome in the world). The aquatic biome can be broken down into two main regions, i.e., (1) freshwater region and (2) marine region. The ever-increasing ecological, social and economic issues alongside a more extensive scope of current research, the utilization of bioactive constituents from natural origin turned out to be more beneficial and acceptable. This growing trend among researchers is due to the easy accessibility and fewer side effect of algal-based bio-resources. Moreover, the exploitation of such natural sources also offers a variety of high-value products which are highly efficient, cost-effective and greener. Considering the immense essence of excellence of algal biome, the principle of “going green” has sifted this alternative search towards eco-friendlier, recyclable and sustainable materials with an overall higher cost-effective ratio benefit. In this context and as discussed above, words like renewable, degradable, and recyclable are emphasized in growing environmental awareness [7,8]. The marine-based sources with high bioactive efficacy have noteworthy advantages over synthetic sources. In this context, natural sources based integrated transition to meet bio-economy challenges and opportunities have following justifications [9]:

(i) to safeguard the natural ecosystem
(ii) to circumvent or diminish the current price hike
(iii) to provoke awareness on the worldwide climate issues
(iv) to stimulate the greener development of regional and rural areas
(v) to diminish the activities which cause greenhouse gasses emission
(vi) to strengthen and diversify the bio-renewable-based energy sources
(vii) to circumvent an over dependency on petrochemicals and/or petro-sources
(viii) to decrease/circumvent the over-consumption of the oil, gas, coal and other potential minerals

**Figure 1:**
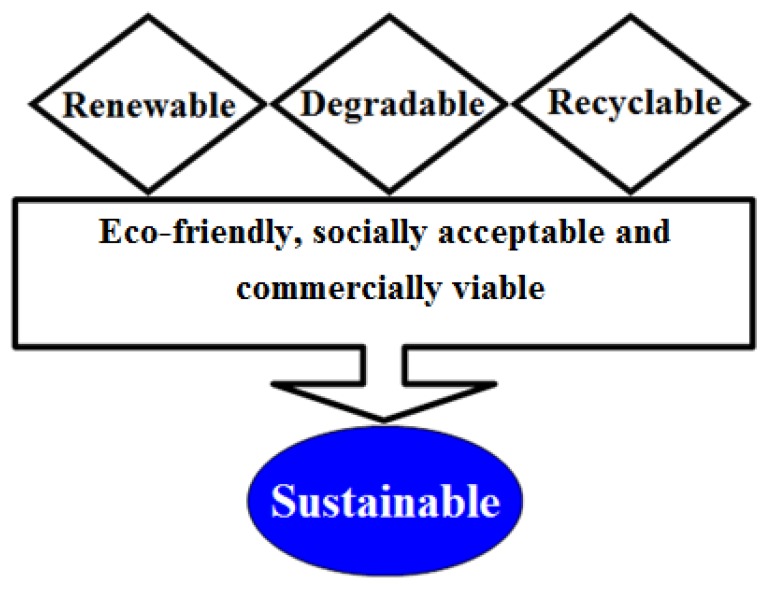
Concept of “sustainability”. Adapted with permission from Iqbal, [7].

The development of distinctive methodologies and/or strategies are in practice for the improvement of cutting-edge bio-based platforms which support green agenda. Thus, the synergistic use of natural materials such as algal-based sources in combination with green technologies is mandatory to establish a sustainable production of value-added products with multifunctional potentialities. The key scientific advances in green biotechnology, as a set of green principles, have extraordinary potential to abolish the generation of wasteful protection and de-protection steps [7]. Aiming to develop either a methodology or products which are genuinely green in nature, pose fewer or no side-effects, and comes under the sustainability concept, the research community either from academia or industry could, and should, consider the green principles from the green agenda [9]. The sustainability concept is shown in Figure 1 [7].

## 3. Algal Biome as A Prolific Source of Bioactive Compounds

**Figure 2:**
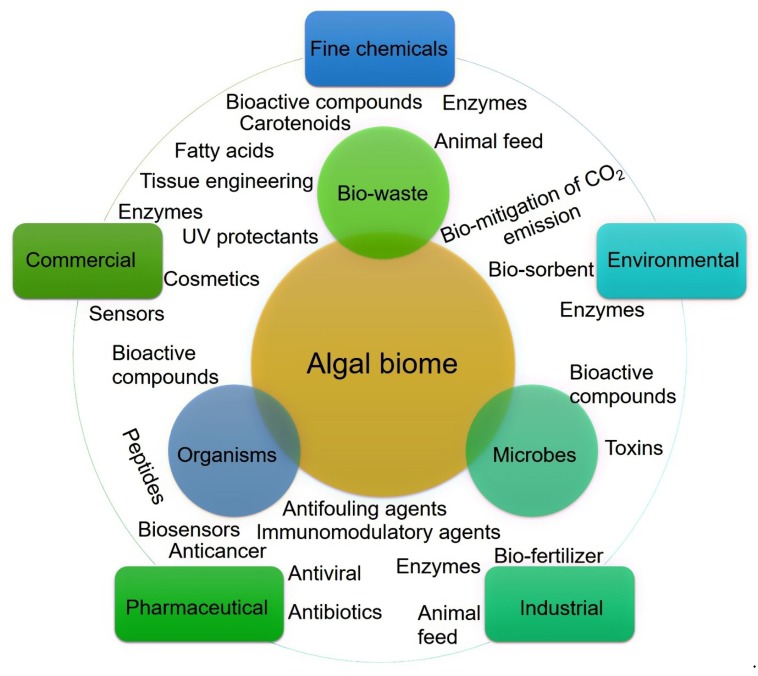
Algal biome as a prolific source of value-added products. The diversity of bioactive substances produced in the marine environment and their potential application routes.

*The Primordial Soup Theory* suggests that life began in a body of water, possibly a pond or ocean. Most of the Earth’s microbial diversity is found in the ocean, which ultimately directs an enormous number of bioactive substances [10]. The ocean is the largest marine biome, and within the ocean, algal strains are characterized by high biodiversity with notable potentialities for various applied purposes, for instance, major routes of applications are shown in Figure 2. Algae are a group of photosynthetic organisms and usually classified as (1) red (Rhodophyceae); (2) brown (Phaeophyceae), and (3) green (Chlorophyceae) [9,11]. More specifically, red algae are a group of multicellular eukaryotic organisms that possess chlorophyll and phycobiliproteins. Their red color appearance is precisely due to the high phycoerythrin content.

The brown algae, comprising the class Phaeophyceae, are a large group of marine multicellular algae, including many seaweeds. Its size varies with a broader range of forms. Some brown algae are just a few centimeters long, while others can be up to 60 m in size with a high level of biodiversity. For instance, *Macrocystis pyrifera*, commonly known as giant kelp or giant bladder kelp that belongs to the phylum Ochrophyta, may reach 60 m (200 ft.) in length and forms prominent underwater masses. From the compositional viewpoint, the brown algae are rich in carbohydrates which contributes more than 50% of their total dry biomass. Moreover, the absence of lignin constituents makes them highly favorable candidates to access their carbohydrate (sugar) contents without any pre-treatment process, which is a highly desirable requisite in plant-based materials. Keeping this mind, several brown algae, for instance, *Sargassum baccaularia* and *Sargassum siliquosum* have been cultivated at a larger scale for numerous applications, e.g., alginate extraction [12]. Compared to other algae (green algae), brown algae have also been exploited as a feedstock for bio-based fuels—so-called “bio-fuel or bio-ethanol”—subject to its overall productivity and high yield [13]. Some of its members such as Ectocarpus have been extensively used as a model organism in genomics [14]. The blue-green algae also are known as Cyanophyceae or Cyanophyta are microscopic organisms that are capable of growing in both marine and fresh water. They are categorized in diatoms (*Bacillariophyceae*), green algae (*Chlorophyceae*) and golden algae (*Cyanophyceae*) [11,15]. Table 1 summarizes various algal strains as a prolific source of bioactive compounds.

## 4. Conventional vs. Non-Conventional Extraction Techniques

Based on literature evidence, several types of conventional extraction techniques have been well exploited for the extraction of bioactive compounds from different materials, including marine sources. The existing conventional/classical extraction techniques include: (1) hydro-distillation which is further divided into three categories i.e., (i) water distillation; (ii) water and steam distillation and (iii) direct steam distillation; (2) Soxhlet extraction; (3) maceration; (4) percolation; (5) infusion, (6) decoction; and (7) hot continuous extraction, etc. [43,44,45]. The extraction of organic compounds, including pesticides, polycyclic aromatic hydrocarbons, and phenols from different matrices (soils, sewage sludges, vegetables, plants), has historically been carried out by using Soxhlet extraction [43,46]. However, many of the techniques mentioned above are strongly dependent on various influencing parameters such as extracting power of solvents, sample size, and concentration, etc. Owing to the fact that Soxhlet extraction is solvent-based (mostly harsh ones), often requires pre-digestion by acids and is time-consuming its current place in modern extraction methodology is limited. In addition, the crude extracts in many cases are subjected to preliminary fractionation and/or purification either by solvent fractionation and/or partition. In other cases, a multistep procedure in maceration, and heavy consumption of water and energy in hydro-distillation also poses serious concerns. Other major drawbacks/limitations of conventional extraction techniques include long extraction periods, the need for high purity solvents, evaporation of the huge amount of solvent, low extraction yield, selectivity and thermal decomposition of thermolabile ingredients, etc. [1]. To tackle the gaps and drawbacks of conventional techniques, new alternative and promising non-conventional (modern) extraction techniques (EAE, SFE, MAE, PLF among others) have been proposed and well reported in the recent literature. Most of these extraction techniques are considered “green” in nature [47], as they comply standards set by the U.S. Environmental Protection Agency (EPA). As compared to conventional extraction techniques, the major advantages of non-conventional extraction techniques include eco-friendlier processing conditions, no or less use of hazardous chemicals, safer auxiliary solvents, use of water, and energy efficiency, reduced formation of derivatives, use of renewable feedstocks, overall cost-effective ratio, facile preparatory steps, higher efficacy, prevention of degradation, and avoidance of protection and deprotection steps [1]. The abovementioned promising non-conventional (modern) extraction techniques are further discussed and elaborated in the following section.

## 5. Extraction Methodologies for Bioactive Compounds

### 5.1. Supercritical-Fluid Extraction (SFE)

SFE epitomizes an important green extraction process which is being used for the extraction of high-value bioactive compounds, e.g., pigments and fatty acids, in recent years [47]. It also seems that SFE addresses the main drawbacks of traditional extraction techniques [47,48]. For instance, some of the drawbacks associated with these techniques include their long extraction time, high energy consumption, and waste generation. Besides, conventional techniques are limited in extraction specificity, then purification steps are required in order to isolate the bioactive metabolites of interest [49,50], whereas, SFE shows great extraction selectivity, short processing times, and a low degradability of the extracted product [51,52,53], without the use of non-food grade solvents [54]. SFE uses supercritical fluids, which above their critical point exhibit liquid-like characteristics such as solvent power, and negligible surface tension, etc. as well as gas-like features such as enhanced transport properties [55]. Moreover, SFE requires minimal solvents as compared to other extraction techniques, while it has a broad application for different bioactive compounds [56]. The thermodynamics and heat transfer properties of carbon dioxide (CO_2_) make it the preferred solvent for SFE-based extraction processes [52,57]. Several other features such as its non-toxic nature, chemical inertness, non-flammability, overall cost-effectivenes, facile availability, and environmental friendliness also represent major factors favoring the choice of CO_2_ as SFE solvent [52,58,59]. It also possesses other advantages such as a low critical point (31 °C, 73 bar). Beyond its critical condition, CO_2_ acquires physicochemical characteristics somewhere between those of a gas and a liquid, showing similar viscosities, intermediate diffusivities, and high density enhancing its penetration into materials [56,60]. In addition, the polarity of CO_2_ can be modulated by the use of co-solvents such as ethanol, increasing the extraction yields of polar compounds [61,62]. Combinations of parameters (temperature, pressure, and co-solvent) are necessary to extract a target compound efficiently. For this purpose, experimental designs are being frequently used, such as the Taguchi method, especially for the evaluation of several process factors at a time with a minimal number of experimental runs (orthogonal array) [63,64]. However, the extract composition is greatly affected by the input conditions of pressure, temperature and co-solvent flow, predictive models, could be used to approach input values about the desired output [47].

Despite the potential of this technique, its usefulness to extract high-value bioactive compounds from algal-based sources strongly depends on the type of compounds to be extracted. Mendiola et al. [65] used SFE to isolate an extract of green microalgae, i.e., *Dunaliella salina* in the presence of CO_2_ at 314 bar and 9.8 °C. The obtained *D. salina* extract displayed notable antimicrobial activity against *Escherichia coli*, *Staphylococcus aureus*, *Candida albicans*, and *Aspergillus niger*. According to the authors, this was probably due to the presence of indolic compounds, PUFAs, and compounds related to carotene metabolism, such as β-ionone and neophytadiene in the microalga extract. Likewise, other bioactive compounds such as vitamin E and carotenoids, among others, have been extracted from algal-based matter. For instance, a statistical approach, i.e., central composite circumscribed design (CCCD) has been employed to optimize an extraction process based on SFE at pilot scale to obtain fractions highly enriched in vitamin E from *Spirulina platensis* [66,67]. The authors have also achieved a tocopherol enrichment of more than 12 times the initial concentration of tocopherol in the raw material by extracting with neat CO_2_ at 361 bar and 83.3 °C. Other target bioactive compounds, such as diolefins, have been extracted from *Botrycoccus braunii* cells by SFE [68].

#### Schematic Workflow of SFE

**Figure 3:**
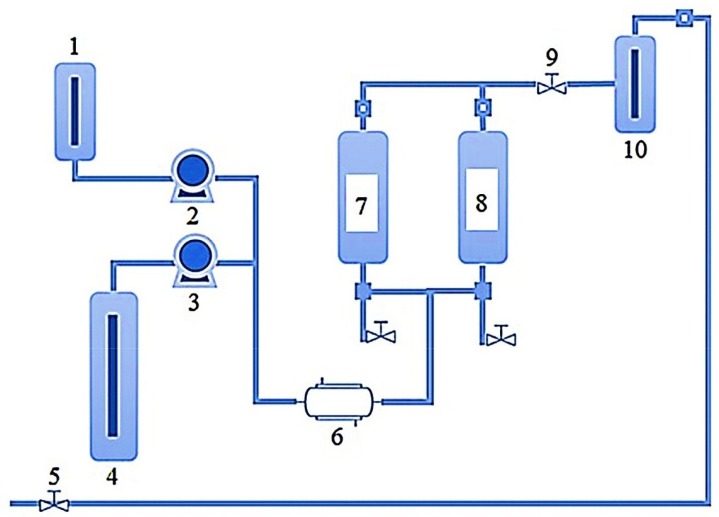
A schematic representation of SFE equipment and working conditions [where: 1: Co-solvent vessel; 2: Co-solvent pump; 3: CO_2_ pump; 4: CO_2_ tank; 5: Manual BPR; 6: Heat exchanger; 7: Extraction vessel 1; 8: Extraction vessel 2; 9: Automated BPR; and 10: Collection vessel]. Reproduced from García-Pérez et al. [47], with permission from Elsevier.

A schematic representation of SFE equipment and working conditions are shown in Figure 3. The SFE workflow in the extraction of bioactive compounds from marine sources involves several parameters, for instance, solvent type, temperature, pressure, sample composition, and concentration, sample quantity, the particle size of the sample, dispersing agents, etc. Among all, the supercritical fluidic dynamics have a strong influence related to the solubility of the target bioactive compounds. This also changes with reference to the extraction temperature and pressure. Optimal sample size also contributes significantly to the higher extraction yield and ultimately strengthens the overall cost-effectiveness of the process as per green agenda. As shown in Figure 3 [47], the SFE equipment comprises following components/parts i.e., (1) a tank (sometimes also called the co-solvent vessel) containing the mobile phase; (2) a pump that pressurizes the mobile phase; (3) a pump that pressurizes the gas (CO_2_); (4) a gas (CO_2_) tank; (5) a manual backpressure regulator (BPR); (6) heat exchanger; (7) extraction vessel 1; (8) extraction vessel 2; (9) automated BPR; and (10) collection vessel. A stepwise workflow of the SFE system is based on: (1) dynamic mode; (2) static mode; or (3) some combination of both modes.

### 5.2. Microwave-Assisted Extraction (MAE)

MAE is another unique approach that yields high titers of quality extract and value-added biological compounds of industrial interest. As compared to traditional solvent-based extraction methods, MAE, being environmental friendlier, has several advantages such as facile operational conditions, minimal solvent use, non-corrosive solvents, short extraction period, overall low consumption ratio for energy and temperature, and inhibits degradation of thermo-labile compounds [69,70]. Therefore, MAE is widely used in several industrial practices for the extraction of high-value bio-active phenolic compounds, phytonutrients, functional foods, and active pharmaceutical grade constituents from biomaterials [47,52,71,72,73]. Despite the fact that algal biome as a prolific source of bioactive compounds, as discussed above, only a few reports on the use of MAE of compounds such as alkaline galactans, carrageenans, and agar from seaweeds and other algal sources [74,75,76,77,78,79]. For instance, Rodriguez-Jasso et al. [78] used the MAE approach to extract sulfated polysaccharides (fucoidan) from brown seaweed, i.e., *Fucus vesiculosus.* Aiming to obtain maximal extraction yields, numerous parameters such as pressure (30–120 psi), extraction time (1–31 min), and alga/water ratio (1/25 to 5/25 g/mL) were evaluated and optimized. Subject to each experimental condition, the alga degradation (%), total sugar yield (%), and SO_3_ content (%) were also determined by Rodriguez-Jasso et al. [78].

#### Schematic Workflow of MAE

In MAE-based extraction system, microwave irradiation is used, which causes motion of polar molecules and rotation of dipoles to heat solvents. This unique feature of MAE promotes efficient transfer of target compounds from the sample matrix into the solvent [80]. The schematic workflow of MAE starts with the homogenization of the samples which are later mixed with a solvent. Regarding the solvent, the MAE technique is very versatile due to the possibility of using numerous solvents such as acetone, acetonitrile, ethanol, methanol, and dichloromethane, with different polarity indexes. Next, the samples are placed in a safe chamber and irradiated with non-ionizing electromagnetic waves of a frequency at more than 2000 MHz for a short period. The irradiation period is usually repeated several times with consecutive cooling periods to avoid sample boiling. According to physics at large and microwave theory, in particular, microwaves are comprised on two oscillating perpendicular fields, i.e., (1) an electric field and (2) a magnetic field. The former field is considered responsible for the heating phenomenon [81]. Furthermore, the heating principle is governed by two phenomena, i.e., (1) dipole rotation and (2) ionic conduction [81,82,83]. In most cases, both happen concurrently, and the microwave-based heating has a direct impact on the polarity of the test materials/solvents [83]. In summary, owing to the high moisture level, marine-based sources are excellent candidate materials for the extraction of bioactive compounds using MAE. This is because the moisture contents serve as the main target for microwave heating. Upon microwave heating, the moisture evaporates and generates an enormous amount of pressure which ultimately ruptures the outer cell membranes and facilitates leaching out of the bioactive compounds [83]. MAE equipment comprises four major components, i.e., (1) a microwave generator; (2) a waveguide which is used to propagate the microwaves from the source to the microwave cavity; (3) a sample incubator; and (4) a circulator which allows the microwaves to move only in the forward direction.

### 5.3. Pressurized-Liquid Extraction (PLE)

In recent years, PLE has been considered an excellent technique for the extraction of polar compounds, as compared to other conventional extraction strategies [84]. In 1996, Richter et al. [85] introduced accelerated solvent extraction (ASE) as a new technique to prepare samples and extract high-value compounds by combining elevated temperatures and pressures with liquid solvents. Owing to its diverse working conditions, ASE is also variously known as pressurized-liquid extraction (PLE), pressurized fluid extraction (PFE), enhanced solvent extraction (ESE), and/or high-pressure solvent extraction (HPSE) [86]. Like other green extraction methods, PLE also has several advantages over traditional extraction approaches. For instance, the minimal consumption of organic solvents, and lower extraction time potential of PLE perfectly meet the green agenda which comes under the Green Chemistry and Engineering (GCE) principles [87]. Under GCE terms, PLE has been successfully used for the extraction of bioactive natural products from marine sponges and other natural materials [84,87].

Very recently, Otero et al. [88] used the PLE technique to extract the high-value fatty acids from macroalgae species from the Northwest of Spain, i.e., *Ulva intestinalis*, *Ulva lactuca*, *Fucus vesiculosus*, *Dictyota dichotoma*, *Cystoseira baccata* and *Himanthalia elongate*. The lipid content (%) profile, and fatty acid composition (mg/g) of four brown species of macroalgae (*F. vesiculosus*, *D. dichotoma*, *C. baccata* and *H. elongata*) and two green algae (*U. intestinalis*, *U. lactuca*) were determined and ranged from 4.6% to 6.7% (Table 2).

In an earlier study, Shang et al. [89] extracted fucoxanthin from brown algae, i.e., *Eisenia bicyclis* (Kjellman) Setchell, using PLE. A statistics-based experimental design was adopted to process optimize the important variables. First, a Plackett–Burman design (PBD) was used to screen out the most important and six influencing parameters i.e., temperature (°C), ethanol concentration (%), static time (min), pressure (psi), weight of sample (g), and flush volume (%). Following that, a second design, i.e., a central composite design, was used to further optimize and attain the best of the selected factors, i.e., temperature (°C), and ethanol concentration (%) for the highest yielding fucoxanthin extraction [89]. Anaëlle et al. [90] performed different PLE extraction experiments along with other extraction techniques such as centrifugal partition extraction (CPE), and supercritical fluid extraction (SFE) to extract bioactive phenolic compounds from brown macroalgae using *Sargassum muticum* as a model. In summary, the use of PLE for the extraction of algal-based bioactive compounds has enormous potential and applications are expected to continue to grow in the following years.

#### Schematic Workflow of PLE

Likewise, SFE, the PLE-based extraction processes can also be done in two modes, i.e., dynamic or static mode. The static mode is more frequently used based on commercial equipment availability as compared to dynamic mode. In PLE, the highest recovery of bioactive compounds can be achieved by optimizing some critical parameters. For instance, the most important parameters which can significantly contribute to the product recovery include temperature, pressure, extraction solvent, static time, and a number of cycles. Other parameters such as purge time and flush volume have shown little influence on the final recoveries, so these are usually fixed. Each parameter can be optimized separately or using experimental designs [86]. Some other parameters such as the arrangement of the sample inside the extraction vessel and the collection of the analytes should also be considered based on the target compound. Of course, for highly volatile compound recovery, a cooling step should be included.

**Figure 4:**
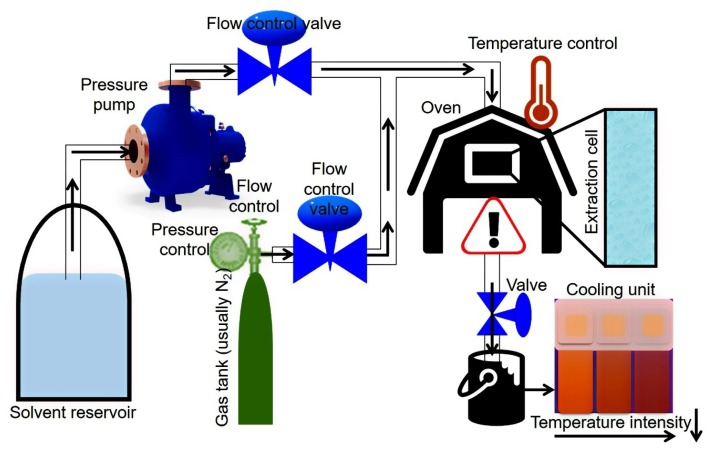
A schematic representation of PLE equipment and working conditions.

A schematic representation of PLE equipment and working conditions are shown in Figure 4. Principally, the PLE-based extraction system comprises a series of solvent reservoirs which are coupled to a high-pressure pump. A gas (usually N_2_) tank and solvent reservoir follow the pressure pump, and both are connected to an oven. Valves control the solvent and gas flow into the extraction cell which is placed inside the oven, each separately, to maintain the pressure inside the system. The extracted sample is collected at the end of extraction system outlet and additionally can also be pumped to a cooling unit for rapid cooling of the resultant extract.

### 5.4. Enzyme-Assisted Extraction (EAE)

EAE of bioactive compounds from numerous sources, including marine ones, has received much attention in recent years. As compared to other reported conventional extraction methods, EAE offers some noteworthy advantages i.e., (1) high selectivity; (2) overall efficacy, (3) rapid extraction, (4) eco-friendly procedures, (5) low-energy consumption, (6) minimal usage of harsh chemicals, (7) maximal yield, (8) low/no wasteful protection/deprotection steps, (9) facile recovery, and (10) process recyclability [73,91,92,93]. In addition, enzyme-based pre-treatments also help induce mass transfer phenomena and ultimately facilitate the release of bioactive compounds and other secondary metabolites in an efficient manner [94]. A range of enzymes including ligninolytic, cellulolytic, and proteolytic enzymes have been extensively used as perfect catalysts. Enzyme-based pre-treatment or catalysis easily causes the breakdown and/or hydrolysis of complex materials on the cell walls and membranes, thus also supporting the recovery of intracellular bioactive constituents which are not easily extractable through conventional extraction methods. This is because the intracellular bioactive constituents are generally intact and compacted in polysaccharides-lignin chains, which limits their extractability [73].

Algal-based seaweeds have been appeared as promising materials to extract bioactive compounds. The presence of complex polysaccharides in the seaweed cell wall, such as alginates and carrageenans, represent physical barriers and reduce the extraction efficacy of general procedures. The EAE-based extraction approach has unique potential to overcome this drawback and facilitate the extraction of bioactive compounds by degrading the cell wall polymers such as alginates. In an earlier study, Barzana et al. [95] presented EAE of carotenoids from *Tagetes erecta*. Under optimized extraction conditions, up to 97% recovery yield of carotenoids was obtained. Various proteases have been used to extract bioactive peptides via hydrolytic reactions [96]. Recently, del Pilar Sánchez-Camargo et al. [97] used EAE in combination with PLE to improve the extraction of phlorotannins from the seaweed *Sargassum muticum*. Enzymatic treatment with proteases and carbohydrases, alkaline hydrolysis and PLE with ethanol:water as extracting solvent have been studied [97]. Under these conditions, values of 21.9%, 94.0 mg gallic acid equivalents g^−1^, 5.018 mg phloroglucinol equivalents g^−1^ and 1.275 mmol Trolox equivalents g^−1^ were obtained for extraction yield, total phenols, total phlorotannins, and TEAC, respectively [97].

#### Schematic Workflow of EAE

**Figure 5:**
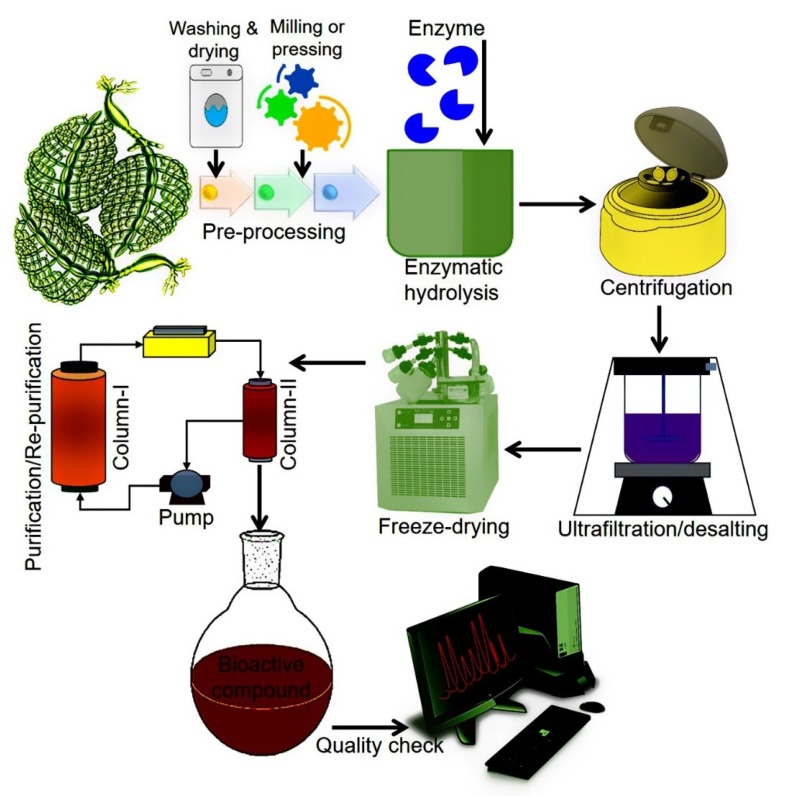
A simplified schematic flow of EAE of algal-based bioactive compounds.

From the GCE perspective, enzymes have been considered ideal biocatalysts with notable potential to assist the extraction of bioactive compounds of natural origin. Principally, EAE facilitates the degradation of cell walls and membranes which is a critical step in the extraction process. This, in turn, increases the cell wall permeability and thus, higher extraction yields of bioactive compounds are achieved [98]. More specifically, EAE is based on two approaches, i.e., (1) enzyme-assisted aqueous extraction (EAAE) and (2) enzyme-assisted cold pressing (EACP) [99]. For efficient and high yield extraction, several factors such as enzyme type and concentration, working pH and temperature, surface area, solid to water ratio, moisture contents, the composition of the test material, the particle size of the samples, incubation, and hydrolysis are considered key factors [1,100,101]. According to GCE, EAE-based extraction of bioactive compounds is documented as an eco-friendly approach because it uses water as a solvent instead of organic chemicals [98]. Figure 5 illustrates a schematic representation of the EAE of bioactive compounds.

## 6. Point-of-Care Applications of Bioactive Compounds

The mechanistic integration of GCE along with the efficient utilization of naturally inspired prolific sources such as algal-based materials and the above-mentioned extraction strategies are mandatory, to establish a sustainable production of high-value bioactive compounds that includes but is not limited to phenolic compounds, tocopherols, carotenoids, organosulfur compounds, phytosterols, fucoxanthin, etc. with different chemical structures e.g., hydrophilic, lipophilic, etc. [102]. GCE offers a wide-ranging set of green principle extraction strategies to obtain products which are genuinely green in nature, pose fewer or no side-effects, and that fall under the sustainability concept. In this context, the research community, either from academia or industry, should consider the green principles for point-of-care applications [9].

**Figure 6:**
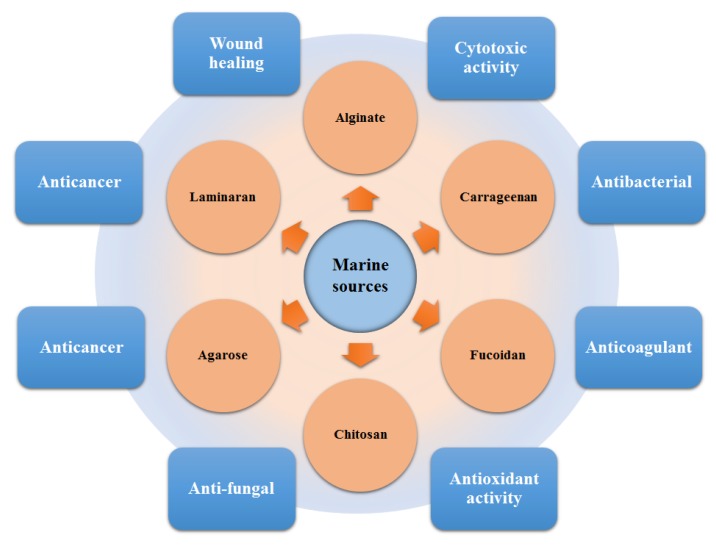
Bio- and non-bio-related applications of marine-based potential sources. Reproduced from Centella et al. [9], with permission from Elsevier.

**Figure 7:**
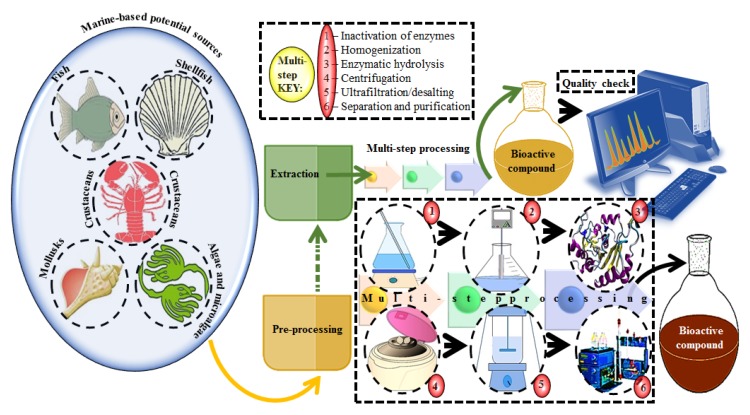
A stepwise illustration to extract and purify bioactive compounds from marine-based potential sources. Multi-step processing key is presented from the pre-processing to purification. Several combinations of chromatographic techniques can be used to achieve high throughput screening and percent purification. For validation purposes, numerous analytical equipment and instrumental techniques can be used to identify and quantify the active fractions of extracted compounds. Reproduced from Centella et al. [9], with permission from Elsevier.

A plethora of marine-derived bioactive compounds with medicinal value such as anticancer, antibacterial, antifungal, antiviral, and anti-allergic agents, etc. are available for a variety of industrial applications at large and pharmaceutical and/or biomedical ones in particular. Marine-derived bioactive compounds are pharmacologically active constituents with great chemical and structural diversity, and thus are considered as potential treatment candidates [103,104,105], thus having great potential to produce high-value therapeutic entities. Several bio- and non-bio related applications of marine-derived bioactive compounds are shown in Figure 6 [9], whereas, Figure 7 illustrates a step by step purification process for bioactive compounds using various marine-based potential sources [9].

### 6.1. Anticancer Potential of Algal-Based Bioactive Compounds

**Figure 8:**
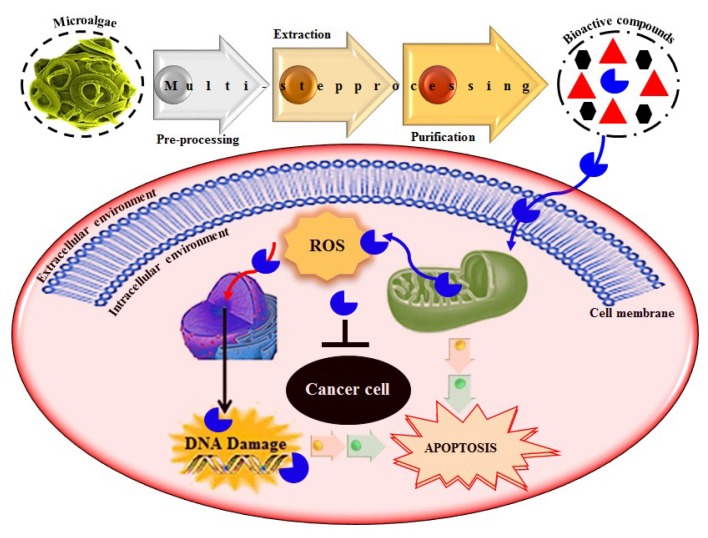
The proposed molecular mechanism bioactive compounds, e.g., fucoidans induced ROS-dependent apoptosis in a cancer cell. Reproduced from Centella et al. [9], with permission from Elsevier.

Based on literature evidence, cancer, in several forms, is recognized as a significant global health-related issue. It is also considered one among the primary leading causes of death. According to a report, in 2012, 32.6 million people were living with cancer while 8.2 million cancer-caused deaths occurred worldwide [9,106]. A broad spectrum of marine-derived bioactive compounds with anticancer potential have been well reported and reviewed [9,107,108]. Cell death can be triggered by three mechanisms, i.e., (1) apoptosis; (2) angiogenesis inhibition and (3) affecting the tubulin-microtubule equilibrium [109]. Most of the available commercial drugs focus on inhibiting any of the abovementioned mechanisms. However, algal-based bioactive compounds have enormous potential to alter several physiological mechanisms, e.g., oxidative stress, inflammation, and carcinogenesis [110]. Further to this unique behavior, some marine-derived compounds, i.e., fucoidans, directly induce cytotoxicity and apoptosis in cancer cells. Thus, there has been growing research interest in the use of fucoidans as an anti-cancer agent in both in-vivo and in-vitro test models [111]. The proposed molecular mechanism of bioactive compounds, e.g., fucoidans-induced ROS-dependent apoptosis in a cancer cell is shown in Figure 8. Anastyuk et al. [112] examined the structural features and anticancer activity in-vitro of depolymerized fucoidan derivatives from the brown alga *Saccharina cichorioides*. The anticancer activities of different concentrations, i.e., 50, 100, 200, and 400 μg/mL of depolymerized fucoidan-based polysaccharides were recorded against the human colorectal adenocarcinoma cell line HT-29. Likewise, in another study by Zhang et al. [113], fucoidan extract isolated from *Cladosiphon navae-caledoniae* Kylin through enzymatic digestion enhanced the anti-cancer activity of chemotherapeutic agents, i.e., cisplatin, tamoxifen or paclitaxel in MDA-MB-231 and MCF-7 breast cancer cells. 

In [113] The authors have also observed that combination treatments enhanced intracellular ROS levels and reduced glutathione (GSH) levels in breast cancer cells, suggesting that induction of oxidative stress was an important event in the cell death induced by the combination treatments. Very recently, Pawar et al. [114] prepared doxorubicin (DOX)-loaded nanoparticles (NPs) using fucoidan and evaluated them as an improved chemotherapy against breast cancer through the immunotherapeutic activity of fucoidan based on an in-vivo model using 4T1 induced tumor-bearing BALB/c mice. Structural characterization and antitumor effects of enzymatically digested fucoidans extracted from the brown alga Kjellmaniella crassifolia have been reported [115]. The enzymatically digested crude extract was further separated into three fractions, i.e., F1, F2, and F3. Based on the composition and structural analyses, F1 was found to have an acetylated galactofucan, F2 consists of fucose, galactose, mannose, and glucuronic acid, while the last fraction, i.e., F3 has two major components, i.e., (1) an acetylated galactofucan and (2) a pure sulfated fucan. The cytotoxicity of all three fractions was tested against murine hepatocarcinoma Hca-F cells in vitro and found a significant inhibition of lump growth in Hca-F-inoculated mice. This also led to upregulated FAS expression in tumor tissues compared to that of the control [115]. A simultaneous administration of fucoidan in combination with a therapeutic agent, i.e., cisplatin, synergistically inhibited lung cancer cell viability by inducing apoptotic responses, including upregulating cleaved caspase-3 and poly (ADP ribose) polymerase (PARP) expression [116]. The efficacy of low-molecular-weight fucoidan as a supplemental therapy in metastatic colorectal cancer patients has been studied by Tsai et al. [117], using a double-blind, randomized controlled trial.

### 6.2. Antibacterial Potential of Algal-Based Bioactive Compounds

In recent years microbial-based serious infections and/or the antimicrobial resistance (AMR) or multidrug resistance (MDR) issues that constantly affect human health have become a worldwide concern [118]. With ever increasing scientific knowledge and social awareness, now the people are more concerned about the AMR/MDR issue. This scenario is even worse as there has been a significant increase in the appearance of AMR/DMR strains that limits the overall effectiveness of several in practice commercial products, including antibiotics [7]. Owing to this increasing consciousness and growing demands of legislative authorities, drug manufacturers, to eliminate AMR/DMR issues in healthcare facilities and possibly reduce pathogenic infections, consider the development of novel anti-microbial active compounds/constituents to be a potential solution to such a problematic issue. Among the potential causes, below are some possible explanations for an increased incidence of AMR/MDR [118]:

(1) The genetic transformation from strain to strain.
(2) Biofilm matrix forming potential of several strains.
(3) Efflux pumps and other outer membrane structural variations.
(4) Enzyme-mediated resistance against, in practice, antimicrobials.
(5) Enhanced level of metabolic activity within the biofilm structure.
(6) Lower/no perfusion of antimicrobial agents through the biofilm matrix.
(7) Adaptability and interaction between antimicrobial agents and biofilm matrix.
(8) Excessive/useless consumption of in practice antimicrobials in a random order.
(9) Genetic variation and adaptability against excessive antimicrobials exposure.

The scenarios mentioned above stimulate the search to develop new types of antimicrobial agents using various sources, including marine-derived bioactive compounds. Therefore, researchers around the globe are valorizing algal-based sources to attract the considerable attention of both academia and industry, especially in the biomedical, and other health-related sectors.

**Figure 9:**
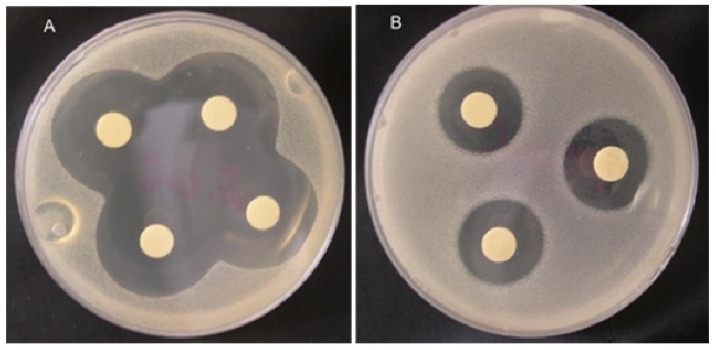
Inhibition zones obtained by the methanolic extract of *Corallina officinalis* against (**A**) *E. coli* and (**B**) *E. faecalis* on Tryptic Soy Agar (TSA) media. Adapted from Taskin et al. [119], an open-access article distributed under the terms of the Creative Commons Attribution License 4.0.

Taskin et al. [119] isolated methanolic extracts of six marine algae belong to the Rhodophyceae (*Corallina officinalis*), Phaeophyceae (*Cystoseira barbata*, *Dictyota dichotoma*, *Halopteris filicina*, *Cladostephus spongiosus f. verticillatus*) and Chlorophyceae (*Ulva rigida*) from the North Aegean Sea (Turkey). The isolated extracts were tested against three Gram+ strains, i.e., *Staphylococcus aureus*, *Micrococcus luteus* and *Enterococcus faecalis* and three Gram− strains, i.e., *Escherichia coli*, *Enterobacter aerogenes*, and *E. coli* O157:H7 using an in-vitro model. Among all tested extracts, *C. barbata* has shown a broader activity spectrum against all the test organisms (Figure 9) [119]. In consideration of the emerging or re-emerging resistance of microorganisms to existing antibiotics, in an earlier study, Bansemir et al. [120] screened 26 species of cultivated seaweeds to investigate the antibacterial activities of their respective extracts. For this purpose, the extracted were prepared using dichlorometane, methanol, and water and tested against five fish-pathogenic bacterial strains, i.e., *Aeromonas salmonicida*, *Aeromonas hydrophila*, *Pseudomonas anguilliseptica*, *Vibrio anguillarum*, and *Yersinia ruckeri*. According to the authors [120], the dichloromethane-assisted extracts of around six out of 26 algal species that includes *Asparagopsis armata*, *Ceramium rubrum*, *Drachiella minuta*, *Falkenbergia rufolanosa*, *Gracilaria cornea*, and *Halopitys incurvus* showed strong antibacterial activities when tested via an agar diffusion assay. The synergistic effect of fucoidan (a sulfated polysaccharide that is primarily extracted from brown seaweeds) with antibiotics, i.e., ampicillin and gentamicin, has also been evaluated against oral pathogenic bacteria either alone or in combination with antibiotics, via the broth dilution method and chequerboard and time-kill assays [121]. This list further grows, and a comprehensive overview of pharmacological mechanisms and applications of marine algae with reference to antibacterial derivatives has been reported by Shannon and Abu-Ghannam [122].

### 6.3. Antiviral Potential of Algal-Based Bioactive Compounds

In recent years, a huge number of viral infectious diseases have emerged or (re)-emerged. In practice current antiviral therapeutics, e.g., oseltamivir and zanamivir, etc. are facing increasing problems with resistance development. Oseltamivir is a selective antiviral prodrug which is used to tackle influenza virus, whereas, zanamivir is an inhibitor of neuraminidase used in the treatment of common flu and prophylaxis of virus A and B. Engineering efficient antiviral drugs with potent activities against a wider spectrum of viral pathogens is difficult because viruses use the host’s cells to replicate [123]. Therefore, researchers, around the globe, are working to extend the range of antivirals to other families of pathogens. Owing to the ever-increasing drug resistance, there is an urgent need to develop novel formulations in a range of contexts to tackle various viral infections. Furthermore, the constantly changing genetic makeup of viruses may alter or induce the viral resistance against several in-practice treatment strategies [124]. Spontaneous or intermittent mechanisms are mainly responsible for viral resistant throughout the antiviral treatment. In an earlier study, Herlocher et al. [125] isolated three type A influenza viruses, each of which has a distinct neuraminidase-gene mutation and is resistant to the neuraminidase inhibitor oseltamivir. Likewise, immunocompromised patients, who received oseltamivir for “post-exposure prophylaxis” are also at higher risk of resistance [126].

Therefore, new antiviral active principles are required, especially from sources that do not constitute or are directly exposed to viral pools. Microalgae have consequently received more attention as a potential source of antiviral agents [127,128,129]. The antiviral activities of extracts of blue-green algae, i.e., *Lyngbya lagerheimeii* and *Phormidium tenue* against human immunodeficiency virus (HIV) along with the protective potentialities for human lymphoblastoid T cells from the cytopathic effect of HIV infection has been reported in earlier studies [130]. A new class of HIV inhibitors called sulfonic acid containing glycolipids, were isolated from the extract of blue-green algae and the compounds were found to be active against HIV [131]. So far, various reasons have been postulated for this activity such as the fact blue-green algae-based cyanoviridin–N inactivates HIV strains and inhibits cell to cell and virus to cell fusion [132]. In other studies, it has also been reported that a novel sulfated polysaccharide, i.e., calcium spirulan (Ca-SP), selectively inhibits the entry of enveloped virus (herpes simplex, human cytomegalovirus, measles virus) into the cell [133,134,135]. Dey et al. [136] reported multiple antiviral activities of cyanovirin-N by blocking the HIV type 1 gp120 interaction with CD4 and coreceptor and inhibition of diverse enveloped viruses. Likewise, red-algae such as *Porphyridium* also produce a sulfated polysaccharide which is able to inhibit viral infection by preventing adsorption of *Herpes simplex* viruses’ types 1 and 2 (HSV 1, and 2), and *Varicella zoster* viruses into the host cells and/or by inhibiting the production of new viral particles inside the host cells [137]. However, the exact action mechanism of antiviral activity of algae extracts and/or algal-based bioactive compounds is not yet fully discovered.

## 7. Research Gaps and Outstanding Questions

A plethora of information is available on naturally occurring bioactive compounds with medicinal potential as antimicrobial, anticancer, antifungal agents, etc. However, many critiques including the distribution profile, safety clearance, in vivo exploitability, GCE-based extraction processes, and yield concerns remain unanswered and need to be addressed in future studies. Despite current biotechnological advancements, the above-discussed concerns, with special reference to marine-derived bioactive compounds, are still at early stages. Therefore, extensive scientific research with proven exploitability following a track record of employability is much needed in this particular line of research. Also, many other questions are yet outstanding and thus pose a big research gap that must be tackled comprehensively:

(1) Is there any significant limitation to judge the proper utilization of marine sources?
(2) Is there any negative impact on the ecosystem subject to the exploitation and application of marine sources?
(3) Is there any approach to limit the dispersion profile and stable the bioactivity profile during extraction?
(4) Is there any tactic or approach to limit the yield and stability variance when extracted from the same or multi-marine sources?
(5) Is there any way to solve the particle size and composition dependent efficacy of the sample extract?

## 8. Concluding Remarks and Future Recommendations

In summary, biologically active constituents, so-called “bioactive compounds”, regardless of the source, will become the norm, not the niche, in the near future. Furthermore, the ever-increasing scientific knowledge and process awareness on the green extraction techniques, offers both a deep insight and attention in the field of the marine-derived bioactive compounds with the following futuristic viewpoints i.e., (1) to design novel extraction processes based on GCE; (2) to explore untouched marine sources with hidden medicinal values; (3) to illustrate workflows and process mechanisms following standard principles; (4) to investigate chemical and structural interactions between different types of active constituents from the same and multi-source; (5) to elucidate the intermediate interactions of bioactive compounds with available drugs; (6) to optimize existing and/or develop new strategies to obtain high yields, etc. As highlighted above, the overall quality and bioactivity of the target compounds strongly depend on different aspects related to the sample preparation to extraction and final separation. In order to obtain maximum product yield, it is equally important to consider all the influential parameters as highlighted in each respective extraction section. For induced yield purposes, all those influential factors should be optimized.

Given the long-term interest in socially acceptable, sustainable and environmentally friendlier extraction methodologies, the versatility, no or lesser side-effects, and high-level bioactivity, algal-based bioactive compounds are likely to remain the subject of intensive research investigations in different sectors of the modern world. Moreover, this could also help in revolutionizing and widen the use and applicability of these naturally occurring rich and novel wealth for numerous health benefits for humans and animals, alike.

## Figures and Tables

**Table 1:** Algal biome as a prolific source of bioactive compounds.

*Algal* spp.	Bioactive Compound	Structure	Methodology	Reference
*Dunaliella salina*	Carotenoids (β-carotene), Chlorophyll a and b	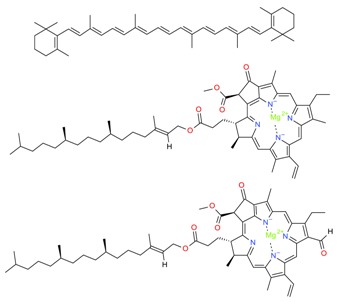	SFE, UAE	Macías-Sánchez et al. [16]
*Pseudoalteromonas phenolica*	MC21-A	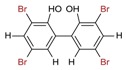	Solvent extraction methanol	Isnansetyo and Kamei [17]
*Chlorella vulgaris Scenedesmus quadricauda*	Antioxidant polysaccharides (sulfated polysaccharides)	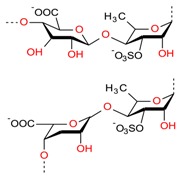	EAE	Mohamed [18]
*Skeletonema marinoi*	Anticonvulsant inosine	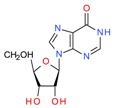	Solvent extraction	Brillatz et al. [19]
*Chlorella vulgaris*	Carotenoids (β-carotene), Chlorophyll a and b	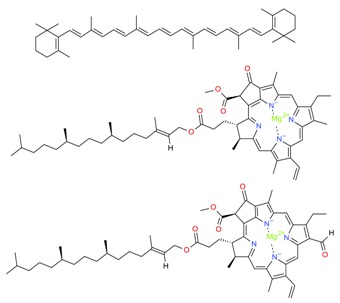	SFE	Safi et al. [20]
***Haematococcus pluvialis***	**Astaxanthin**	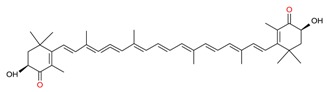	SFE	Li et al. [21]
***Phormidium valderianum***	**Anatoxin-a**	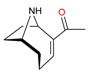	SFE	Chatterjee and Bhattacharjee [22]
***Scenedesmus obliquus***	Lutein	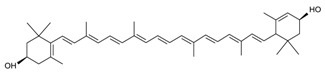	Solvent extraction, UAE	Chan et al. [23]
*Gyrodinium impudium*	Sulfated polysaccharide	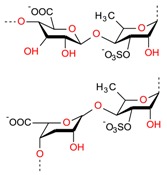	Solvent extraction	Kim et al. [24]
***Nannochloropsis* spp.**	Carotenoids (β-carotene), Chlorophyll a and b	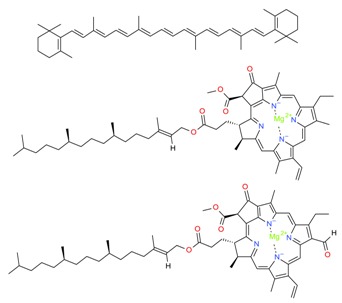	UAE	Parniakov et al. [25]
*Chlorella stigmatophora Phaeodactylum tricornutum*	Crude polysaccharide extracts	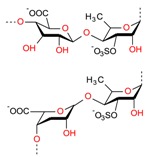	Lyophilized	Guzmán et al. [26]
*Scenedesmus* sp.	Lipids	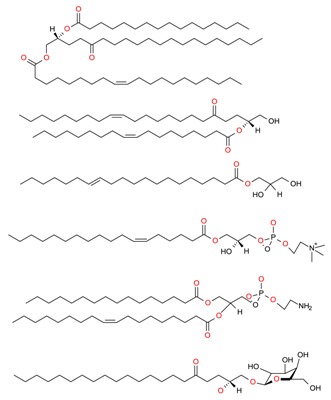	SFE	Taher et al. [27]
*Nannochloropsis oculata*	Lipids	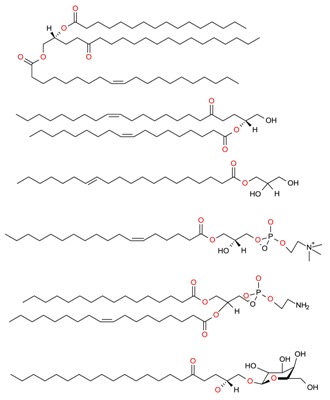	UAE	Adam et al. [28]
***Spirulina platensis***	β-carotene	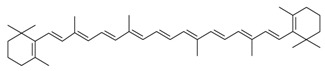	UAE	Dey and Rathod [29]
***Fucus vesiculosus***	Fucoidan	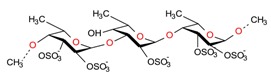	MAE	Hahn et al. [30], Cumashi et al. [31]
***Streptomyces* sp.**	Bahamaolides A and B	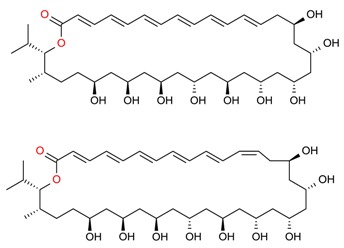		Kim et al. [32]
***Chlorella vulgaris*, *Scenedesmus dimorphus*, and *Nannochloropsis* sp.**	Lipids	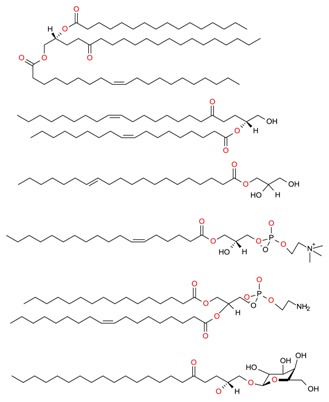	EAE	Liang et al. [33]
***Palmaria palmata***	L-Ascorbic acid Glutathione	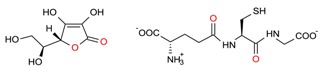	EAE	Wang et al. [34]
***Sargass*** ***um muticum***	Phenolic compounds	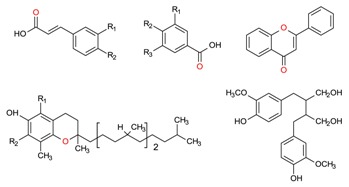	EAE, MAE	Pérez et al. [35]
***Laminaria* and *Saccharina species***	Laminarin	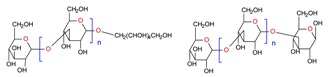	Solvent extraction	Kadam et al. [36]
***Dunaliella salina***	Carotenoids (β-carotene)	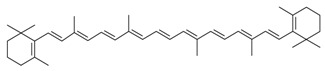	PLE	Herrero et al. [37]
***Phaeodactylum tricornutum***	Fucoxanthin	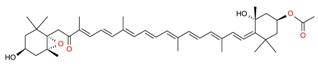	PLE	Kim et al. [38]
***Nannochloropsis* sp.**	Lipids	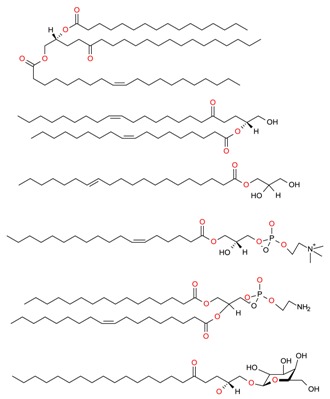	EAE	Zuorro et al. [39]
***Chlorella vulgaris***	Lipids	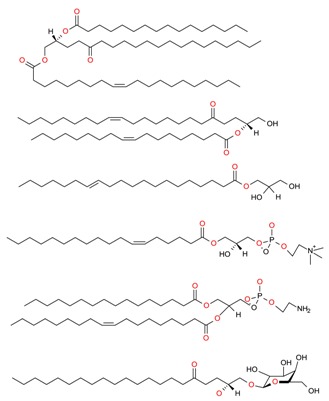	MAE and SFE	Dejoye, et al. [40]
***Chlorella vulgaris***	Lutein	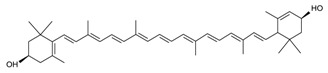	UAE	Deenu et al. [41]
***Neochloris oleoabundans***	Carotenoids (β-carotene)	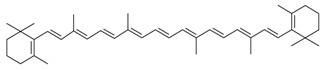	PLE	Castro-Puyana et al. [42]

**Table 2:** Lipid content (%) and fatty acid composition (mg/g) of six algae species (dry weight).

FA	FA (C:U)	RT (min)	*F. vesiculosus*	*C. baccata*	*H. elongata*	*D. dichotoma*	*U. lactuca*	*U. intestinalis*
FA content (mg/g algae)	FA 14:0	9.802	11.09 ± 0.19	5.13 ± 0.12	1.72 ± 0.04	3.01 ± 0.37	1.78 ± 0.22	1.96 ± 0.23
	FA 15:0	10.774	0.31 ± 0.03	N.D	0.17 ± 0.03	N.D	0.18 ± 0.02	0.08 ± 0.00
	FA 16:1	11.789	0.98 ± 0.22	2.19 ± 0.04	0.56 ± 0.01	1.16 ± 0.05	0.16 ± 0.00	0.16 ± 0.00
	FA 16:0	12.078	9.64 ± 0.30	6.80 ± 0.29	5.85 ± 0.14	4.40 ± 0.64	6.09 ± 0.29	6.02 ± 0.22
	FA 18:3	14.900	0.08 ± 0.00	N.D	0.04 ± 0.04	N.D	0.09 ± 0.01	N.D
	FA 18:2	15.304	0.34 ± 0.04	0.16 ± 0.02	0.01 ± 0.00	0.01 ± 0.00	0.05 ± 0.01	0.06 ± 0.00
	FA 18:1	15.507	13.15 ± 1.03	3.09 ± 0.34	0.49 ± 0.09	1.09±0.05	0.47 ± 0.02	0.23 ± 0.01
	FA 18:0	16.041	1.56 ± 0.13	1.65 ± 0.16	1.80 ± 0.04	1.28 ± 0.07	1.68 ± 0.11	2.11 ± 0.08
	FA 20:4	20.549	1.30 ± 0.12	0.62 ± 0.01	N.D	N.D	N.D	N.D
	FA 20:5	20.806	0.36 ± 0.08	0.24 ± 0.01	N.D	0.15 ± 0.03	N.D	N.D
FA total (mg/g algae)			38.83	19.87	10.64	11.09	10.46	10.63
Lipid content by Folch (%)			6.6%	6.7%	6.0%	5.7%	4.8%	4.6%

Lipids were extracted using the Folch method (n = 3). A number of carbon and unsaturation (C:U) status and retention time (RT) of the fatty acid methyl esters (FAMEs) are also included. Results show the mean ± standard error of the mean (SEM) of three experiments. N.D means not detected. Reproduced from Otero et al. [88], an open-access article distributed under the terms and conditions of the Creative Commons Attribution (CC BY) license (http://creativecommons.org/licenses/by/4.0/).
